# Recombinant *Bacillus subtilis* Spores Elicit Th1/Th17-Polarized Immune Response in a Murine Model of *Helicobacter pylori* Vaccination

**DOI:** 10.1007/s12033-015-9859-0

**Published:** 2015-03-18

**Authors:** Małgorzata Stasiłojć, Krzysztof Hinc, Grażyna Peszyńska-Sularz, Michał Obuchowski, Adam Iwanicki

**Affiliations:** 1 Department of Medical Biotechnology, Intercollegiate Faculty of Biotechnology UG-MUG, University of Gdańsk, Dębinki 1, 80-211 Gdańsk, Poland; 2 Department of Medical Biotechnology, Intercollegiate Faculty of Biotechnology UG-MUG, Medical University of Gdańsk, Gdańsk, Poland; 3 Tri-City Animal Laboratory, Medical University of Gdańsk, Gdańsk, Poland

**Keywords:** *Bacillus subtilis*, Spores, *Helicobacter pylori*, Oral vaccine, Th1, Th17

## Abstract

Current progress in research on vaccines against *Helicobacter pylori* emphasizes the significance of eliciting the Th1/Th17-polarized immune response. Such polarization can be achieved by selection of appropriate antigen and adjuvant. In this study, we wanted to check the polarization of the immune response elicited by UreB protein of *Helicobacter acinonychis* delivered by recombinant *Bacillus subtilis* spores upon oral immunization. *B. subtilis* spores presenting fragment of UreB protein and able to express entire UreB in vegetative cells after germination were orally administered to mice along with aluminum hydroxide or recombinant spores presenting IL-2 as an adjuvant. The pattern of cytokines secreted by sensitized splenocytes assessed by the cytometric bead array clearly indicated polarization of the immune response toward both Th1 and Th17 in mice immunized with the use of above-mentioned adjuvants. Obtained result is promising regarding the usage of recombinant spores in formulations of vaccines against *H. pylori* and line up with the current state of research emphasizing the key role of appropriate adjuvants.

## Introduction

*Helicobacter pylori*, a Gram-negative microaerophilic bacterium, infects gastric mucosa of more than half of the world’s population. It is a risk factor of such gastroduodenal diseases as peptic ulcers or gastric cancers [1, 2]. Infection with *H. pylori* induces in gastric mucosa, a strong inflammatory response, characterized by infiltration of neutrophils and T- and B-cells. According to recent results, the major role in this response is assigned to T-helper cells of Th1 and Th17 types, which can be activated in the infected organism with consequent production of IFN-γ, IL-17, and TNF-α (reviewed in [3]). In spite of strong innate and adaptive immune responses elicited by *H. pylori,* infected patients usually fail to clear the infection without appropriate treatment. Current standards for therapy of *H. pylori* infections assume application of multi-drug regimens which can result in significant side effects. Antibiotics used in combination with such drugs as proton pump inhibitors lead to 80–90 % eradication; nevertheless, failures can contribute to development of antibiotic resistance and re-infection [4, 5].

Vaccinations appear to be an attractive alternative to the treatment with antibiotics. Most studies involve the use of urease subunits as antigens in vaccine formulations. Although currently some of such vaccines are used in clinical trials (phase I) [6–8], there is a common agreement that selection of appropriate bacterial antigens is crucial for development of an effective vaccine. One of the promising vaccine candidates is based on multi-epitope DNA vaccine with CpG oligonucleotides and heat-labile enterotoxin LTB as adjuvants [9]. In other trials, mice immunized with *H. pylori* *opiA* gene-encoded construct were able to develop protective immune response when co-administered with IL-2 gene-encoded construct and LTB [10]. In another example, the successful immunization was achieved with CagA, VacA, and UreB proteins of *H. pylori* in different arrangements along with an adjuvant leading to Th1 shift of cellular response [11]. All these examples clearly indicate the importance of appropriate adjuvants in formulations of a vaccine against infections with *H. pylori*. While most studies involved usage of strong immunogens (e.g., bacterial toxins), for this purpose, there is an increasing interest in application of immunomodulatory molecules as adjuvants, e.g., interleukins.

In our recent study, we have shown that recombinant *Bacillus subtilis* spores can also serve as antigen carriers in vaccines directed against *H. pylori*. In our approach, we have used *B. subtilis* spores presenting fragment of UreB protein of *Helicobacter acinonychis* and able to express entire UreB protein in vegetative cells upon germination. Such recombinant spores were able to elicit specific Th1-biased cellular immune response when co-administered with IL-2 presenting spores serving as mucosal adjuvants [12]. *B. subtilis* spores seem to be very interesting platforms for antigen presentation and delivery in mucosal vaccines. Such properties as extreme resistance to harsh environmental conditions (e.g., stomach lumen) as well as the easiness of genetic manipulation make them very suitable for that purpose. This is also reflected in successful application of *B. subtilis* spore-based vaccines in development of protective immunity against such pathogens as *Clostridium perfringens* [13], *Clostridium difficile* [14], *Clostridium tetani* [15], or Rotavirus [16].

Here, we present results indicating that specific cellular immune response elicited in mice by recombinant *B. subtilis* spores delivering UreB of *H. acinonychis* does not only develop upon oral co-administration with IL-2-displaying spores but also used with aluminum hydroxide as adjuvant. Moreover, we show that in case of both formulations, this immune response is of Th1 and Th17 type.

## Methods

### Ethics Statement

This study was carried out in strict accordance with the recommendations in the institutional and national guidelines for animal care and use. The protocol was approved by the Committee on the Ethics of Animal Experiments of the Medical University of Gdańsk (Permit Number: 4/2010). All surgery was performed under isoflurane anesthesia, and all efforts were made to minimize suffering.

### Bacterial Strains

*Bacillus subtilis* strains used in this study were 168 [17], BKH108 (UreB), and BKH121 (IL-2) [12]. BKH108 strain produces spores displaying fragment of UreB protein of *H. acinonychis* as fusion with spore coat protein CotC. The *ureB* gene encoding entire UreB protein of *H. acinonychis* is expressed from *p*_*rrnO*_ promoter. For detailed description, refer to [12].

### Preparation of Spores

Sporulation was induced by the exhaustion method in DS (Difco-Sporulation) medium as described elsewhere [18]. After the final suspension in water, spores were treated at 65 °C for 1 h to kill any residual vegetative cells. The spore suspension was titrated immediately for CFU/ml before freezing at −22 °C. By this method, we could reliably produce 6 × 10^10^ spores per liter of DSM culture.

### Spore Germination

Spores were heat activated at 80 °C for 20 min. Next, serial dilutions were prepared, plated onto LB medium solidified with 1.5 % agar, and incubated overnight at 37 °C. Numbers of obtained colonies were used for calculation of germination efficiency.

### Immunizations

Five groups of eight mice (female, BALB/c, 8 weeks) were immunized by oral route with suspensions of either spores expressing CotC-UreB3 (BKH108) with 3 % Al(OH)_3_, both CotC-UreB3 (BKH108) and CotB-linker-IL-2 (BKH121) (1:1) or control spores (wild-type strain 168). A naive, non-immunized control group was included. Oral immunizations were performed with 1.0 × 10^10^ spores in a volume of 0.2 ml of water administered by intragastric lavage on days 1, 3, 5, 22, 24, 26, 43, 45, and 47. Animals were sacrificed on day 61, and serum samples and spleens were collected.

### Indirect ELISA for Detection of Antigen-Specific Serum

UreB-specific antibodies in saponin extracts of gastrointestinal tracts or sera of immunized animals were detected as previously described [12]. Briefly, plates were coated with 100 μl per well of the specific antigen (2 μg/ml in carbonate/bicarbonate buffer) and left at room temperature overnight. Antigen was UreB-purified protein. After blocking with 0.5 % BSA in PBS for 1 h at 37 °C, samples were applied using a two-fold dilution series starting with a 1/20 dilution in ELISA diluent buffer (0.1 M Tris–HCl, pH 7.4; 3 % (w/v) NaCl; 0.5 % (w/v) BSA; 10 % (v/v) sheep serum (Sigma); 0.1 % (v/v) Triton-X-100; 0.05 % (v/v) Tween-20). Every plate carried replicate wells of a negative control (a 1/20 diluted pre-immune serum) and a positive control (serum from mice immunized intraperitoneally with UreB-purified protein). Plates were incubated for 2 h at 37 °C before addition of anti-mouse AP conjugates (Sigma). Plates were incubated for a further 1 h at 37 °C then developed using the substrate pNPP (para-Nitrophenylphosphate; Sigma). Reactions were stopped using 2 M H_2_SO_4_.

### Isolation of Splenocytes

Mice were sacrificed, and spleens were isolated as previously described [12]. Briefly, mice were sacrificed, and spleens were aseptically removed. The spleens were then perfused with RPMI-1640 (supplemented with 10 % heat-inactivated fetal calf serum, 25 mM HEPES, 2 mM l-glutamine, 1 mM sodium pyruvate, 100 IU/ml penicillin, and 100 mg/ml streptomycin) using 5-ml syringe fitted with 26 G needle to obtain single-cell suspension of splenocytes. The splenocytes suspension was then centrifuged at 300×*g* for 15 min. The RBCs were lysed by hypotonic shock using 3 ml of 0.84 % of sterile NH_4_Cl or ACK lysis buffer for 5 min. The cells were then washed thrice with RPMI-1640 to remove lysed RBCs and NH_4_Cl.

### Activation of Splenocytes

Splenocytes (2 × 10^5^/mL) were cultured in the presence or absence of UreB antigen for 48 h. Samples of supernatants containing released cytokines were collected and stored at −80 °C

### Flow Cytometry

Levels of IL-10, IL-17, TNF, IFN-γ IL-6, IL-4, and IL-2 secreted by sensitized cells were determined by cytometric bead array (CBA) Mouse Th1/Th2/Th17 Cytokine Kit (BD) kit according to manufacturer’s protocol. Measurements were performed using Accuri C6 Flow Cytometer (BD) and results processed with system software. Six technical repeats were done for each animal in the group. Results were statistically evaluated using Student’s *t* test.

## Results

IL-2-presenting spores were shown to have mucosal adjuvant properties [12]. We were interested, whether classical adjuvant, aluminum hydroxide, would also help in eliciting a specific immune response in Balb/c mice upon oral administering of spores of BKH108 (UreB) recombinant *B. subtilis* strain. Our previous studies have shown that efficient immune response to recombinant spores was strictly dependent on the use of an adjuvant. In the current study, we performed immunization cycle using spores of BKH108 strain along with aluminum hydroxide. As a reference in immunizations, we used spores of BKH108 strain mixed with IL-2-presenting spores (BKH121). Prior to immunizations, we tested the influence of aluminum hydroxide on germination of spores of the wild-type and BKH108 strains. We observed lowered germination efficiency of spores incubated in suspension of this compound (Table 1). Animals immunized with both formulations showed no side effects or weight loss associated with immunizations (data not shown).

**Table 1:** Efficiency of germination of spores produced by the wild-type 168 and BKH108 strains

	168	BKH108
H_2_O	100 ± 10^a^	100 ± 13.1^a^
Al(OH)_3_	47.9 ± 5.1	17.98 ± 7.9

^a^Germination of spores suspended in water or 3 % aluminum hydroxide was analyzed as described in “Methods” section. Numbers represent percentages of germinated spores along with standard deviations. Amounts of germinated spores incubated in water were taken as 100. The presented results are averages of three independent experiments

**Fig. 1:**
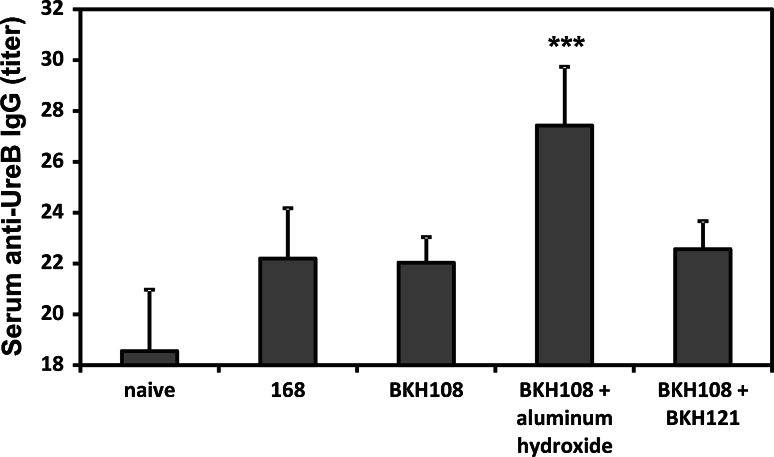
Endpoint titration of IgG antibody to UreB in sera collected from mice upon completion of immunization cycle (day 61). Mice were orally immunized with wild-type 168 or recombinant BKH108 (UreB) and BKH121 (IL-2) spores as described in “Methods” section. *Each bar* represents average of UreB-specific IgG titers in sera of eight animals. *Error bars* represent standard deviation. Statistical significance versus 168 assessed by Student’s *t* test ***p < 0.001

**Fig. 2:**
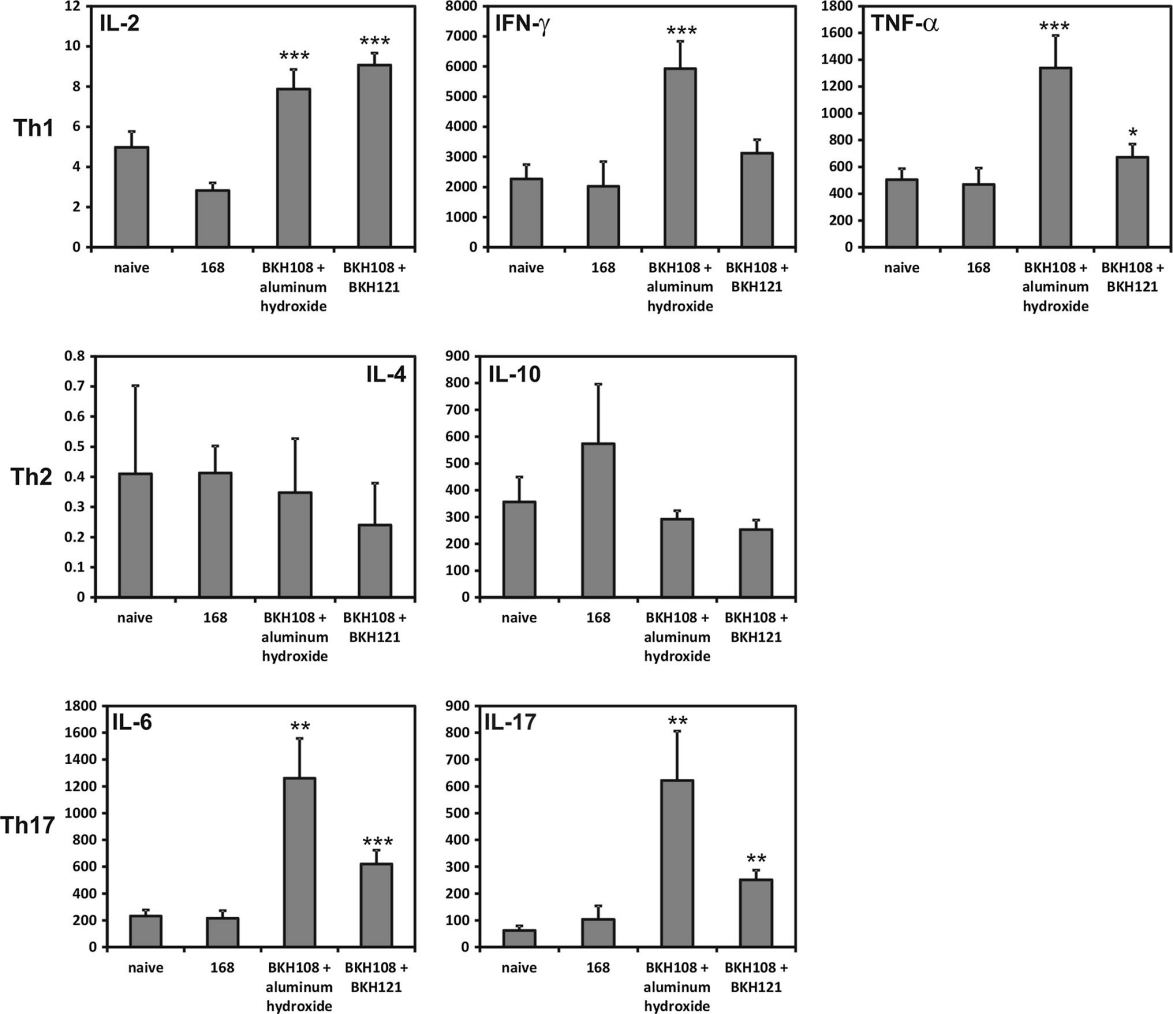
Polarization of immune response is elicited by recombinant spores as assessed by analysis of profiles of cytokines secreted by sensitized splenocytes. Splenocytes were isolated from mice orally immunized with the wild-type 168 or recombinant BKH108 (UreB) and BKH121 (IL-2) spores as described in “Methods” section. Levels of cytokines (pg/ml) indicated in each graph were measures by flow cytometry using the cytometric bead array. *Graphs* are arranged in groups of cytokines characteristic for polarizations of the immune response indicated with Th1, Th2, or Th17. The presented results are averages of cytokines levels secreted by splenocytes isolated from groups of eight animals. *Error bars* indicate standard deviation. Statistical significance versus 168 assessed by Student’s *t* test *p < 0.05, **p < 0.01, ***p < 0.001

Upon completion of immunization cycle, we performed characterization of developed immune response. We observed very low levels of UreB-specific IgG antibodies in sera of immunized animals (Fig. 1) and no such IgG or IgA antibodies in saponin extracts of isolated gastrointestinal tracts (data not shown). Systemic antigen-specific cellular immune response was assessed using the CBA performed on sensitized splenocytes isolated from immunized animals. The composition of the CBA assays used in the research enabled characterizing the type (Th1, Th2, or Th17) of observed immune response as Th1- and Th17-polarized (Fig. 2). In case of both formulations, profiles of secreted cytokines were very similar, but higher amounts of cytokines were observed in samples prepared with splenocytes of animals immunized with BKH108 spores and aluminum hydroxide as adjuvant. Cytokines characteristic for Th1 response: IL-2, IFN-γ, and TNF-α were present in culture supernatants at significantly higher concentrations in case of sensitized splenocytes as compared to control splenocytes (naïve mice or mice immunized with 168 carrier spores). Similarly, levels of IL-17 and IL-6 were significantly higher in samples of supernatants of sensitized cells. At the same time, levels of cytokines characteristic for Th2 response: IL-4, and IL-10, were lowered as compared to control cells.

## Discussion

The development of an efficient vaccine against *H. pylori* infections seems to be a real challenge. In spite of increasing number of studies aimed on creation of such vaccine, still no satisfactory solution has been found. Three critical issues regarding the development of an efficient anti-*H. pylori* vaccine have been proposed, and these are selection of appropriate bacterial antigens, safe and effective adjuvants, and a route of delivery [3].

UreB subunit of *H. pylori* urease has already been used in several studies as an antigen in immunizations against this pathogen. While most of the researches have been done using such recombinant bacteria as *Salmonella typhimurium* [19] or *Lactococcus lactis* [20], few works involved the use of recombinant plants (rice [21], carrot [22], or peanuts [23]) expressing UreB protein. Regarding the route of immunization, oral vaccines seem to be the most interesting since they should lead to the delivery of an antigen directly to the site of infection. Moreover, the easiness of administering of such vaccines is an additional benefit of such vaccines. Due to these facts *B. subtilis* spores seem to be a very good platform for antigen delivery. Indeed, they have already been applied for construction of promising vaccine candidates [13–16].

We wanted to further investigate usefulness of recombinant *B. subtilis* BKH108 spores which deliver the UreB protein of *H. acinonychis*, a close relative of *H. pylori*, as antigen. We decided to use urease subunit B from *H. acinonychis* to avoid any potential patent issues, which may arise in case of use of UreB from *H. pylori*. As the recent progress in the research on vaccines against *H. pylori* suggested the need of application of an appropriate adjuvant, we decided to use for this purpose as a well-known compound, aluminum hydroxide. Aluminum compounds have been used for decades as efficient adjuvants in subcutaneous vaccines, leading to the induction of Th2-polarized immune response [24–26]. Al(OH)_3_ has been shown to induce local decrease in expression of the Th2 cytokine IL-10 and local increase of expression of the Th1 cytokine IL-12 in the stomach in parenteral immunizations [27]. This compound has also been used as adjuvant in mucosal immunizations by intranasal [28, 29] and oral [30] routes. In our previous study, we were able to elicit UreB-specific immune response in mice orally immunized with BKH108 spores only upon co-administering with IL-2 presenting spores [12]. Since IL-2 is a strong immunomodulatory cytokine, it would require very detailed optimization in composing of safe and efficient vaccine formulation consisting of these two types of spores. The decision to use aluminum hydroxide as adjuvant in current study was directed to overcome such issues. Our idea proved to be right, since we obtained efficient UreB-specific immune response in immunized animals. We went further and tried to characterize the polarization of elicited immune response. As shown in Fig. 2, the profile of secreted cytokines suggests the response to be both Th1- and Th17-polarized, with simultaneous inhibition of Th2 polarization. This observation is supported by results of titration of UreB-specific antibodies in the sera of immunized animals. Although we noticed some minimal titers of these antibodies (Fig. 1), we consider them as negligible. It is important to notice that the same pattern of cytokines production was observed for splenocytes isolated from mice immunized orally with BKH108 spores and BKH121 spore, presenting IL-2 (Fig. 2). Obtained polarization of the immune response is promising in light of recent studies on responses of the immune system to infection with *H. pylori*. As reviewed by D’Elios et al. [3], infection with this bacterium leads to activation of both Th1 and Th17 cells with subsequent production of IFN-γ, IL-17, and TNF-α. Moreover, it has been shown that vaccinations promoting Th1 or Th17 immunity are sufficient to protect mice from *H. pylori* [31, 32].

Interestingly, *B. subtilis* spores presenting UreB of *H. pylori* have recently been shown by Zhou et al. to be efficient in protecting of orally immunized mice against infection with this bacterium [33]. This observation does not line up with our previous [12] and current results, since we observed no immune response elicited by BKH108 spores administered without any adjuvant. These discrepancies might partially be explained with differences of UreB used as antigen displayed on the surface of *B. subtilis* spores. While Zhou et al. reported usage of *H. pylori* UreB [33], in our experiments, we used UreB of *H. acinonychis*. This is surprising, up to some extent, because UreB proteins from both microorganisms share 99 % identity of amino acid sequence. Moreover, *H. acinonychis* is an animal pathogen closely related to *H. pylori* and recognized as a useful in vivo model to study *H. pylori* virulence mechanisms [34]. The same study reports production of UreB-specific antibodies in immunized animals [33], but due to the specific way of data presentation (OD_450_ instead of antibody titer), it was not possible to compare it with results obtained in our study. It is also worth mentioning that our results are in agreement with previous studies, in which *B. subtilis* spores presenting antigens elicited strong Th1-biased immune response with no (or very low) humoral response [35, 36].

To conclude, we have shown that usage of aluminum hydroxide in oral immunizations with recombinant spores helped elicit the immune response specific for an antigen presented on the spore surface. Moreover, the polarization of observed response to UreB protein goes along with the current research on vaccines against *H. pylori* suggesting, that formulations basing on recombinant *B. subtilis* spores, supplemented with an appropriate adjuvant, may be useful in protection against infection with this pathogen.
